# Liver and Nonliver-Related Outcomes at 2 Years Are Not Influenced by the Results of the FIB-4 Test and Liver Elastography in a Real-Life Cohort of Patients with Type 2 Diabetes

**DOI:** 10.1155/2021/5582813

**Published:** 2021-03-08

**Authors:** Ivica Grgurevic, Nermin Salkic, Sanda Mustapic, Tomislav Bokun, Kristian Podrug, Srecko Marusic, Dario Rahelic, Tomas Matic, Viktoria Skurla, Ivana Mikolasevic

**Affiliations:** ^1^Department of Gastroenterology, Hepatology and Clinical Nutrition, University Hospital Dubrava, University of Zagreb School of Medicine and Faculty of Pharmacy and Biochemistry, Zagreb, Croatia; ^2^Department of Gastroenterology and Hepatology, University Hospital Centre Tuzla, Tuzla, Bosnia and Herzegovina; ^3^Department of Gastroenterology and Hepatology, University Hospital Split, Split, Croatia; ^4^Department of Endocrinology, Diabetes and Metabolism, University Hospital Dubrava, University of Zagreb School of Medicine and Faculty of Pharmacy and Biochemistry, Zagreb, Croatia; ^5^Vuk Vrhovac University Clinic for Diabetes, Endocrinology and Metabolic Diseases, Merkur University Hospital, University of Zagreb School of Medicine, Zagreb, Croatia; ^6^School of Medicine Osijek, Josip Juraj Strossmayer University of Osijek, Osijek, Croatia; ^7^University of Zagreb School of Medicine, Zagreb, Croatia; ^8^Department of Gastroenterology and Hepatology, University Hospital Merkur, Zagreb, Croatia

## Abstract

**Aims:**

To investigate morbidity and mortality in a real-life cohort of patients with type 2 diabetes (T2D) in relation to prevalence and severity of nonalcoholic fatty liver disease (NAFLD).

**Methods:**

Patients with T2D were referred for assessment of liver fibrosis by the FIB-4 test and liver stiffness measurement (LSM) by vibration-controlled transient elastography (VCTE). Liver steatosis was quantified by the controlled attenuation parameter (CAP). These patients were followed until death or censored date.

**Results:**

Among 454 patients (52% males, mean age 62.5 years, BMI 30.9 kg/m^2^), 82.6% was overweight, 77.8% had fatty liver, and 9.9% and 3.1% had LSM and FIB-4 values suggestive of advanced fibrosis, respectively. During the follow-up period of median 2 years, 106 (23%) patients experienced adverse event (11% cardiovascular) and 17 (3.7%) died, whereas no liver-related morbidity or mortality was observed. Independent predictors of adverse outcomes were age and higher platelet count, while FIB-4, LSM, and CAP were not.

**Conclusion:**

In a cohort of T2D patients, no liver-related morbidity or mortality occurred during 2 years. Our patients probably have low real prevalence of advanced fibrosis which is likely overestimated by LSM ≥ 9.6 kPa. Liver fibrosis may be safely reassessed in the 2 years interval in noncirrhotic patients with T2D.

## 1. Introduction

Type 2 diabetes (T2D) is among the most prevalent conditions today, affecting almost 10% of the adult population worldwide [1]. It is most frequently accompanied by overweight/obesity which represents the causative factor in majority of the patients through the development of insulin resistance. Together with dyslipidemia and arterial hypertension, these factors constitute metabolic syndrome (MetS) which has been recognized as the leading cause of atherosclerosis and subsequent cardiovascular morbidity and mortality.

Patients with T2D are frequently diagnosed with nonalcoholic fatty liver disease (NAFLD), but this condition has not been well appreciated by international guidelines concerning the diagnostic work-up of diabetic patients. However, in the recent years, a significant body of evidence has been accumulated showing very high prevalence of NAFLD in T2D, a combination associated with poor prognosis in terms of adverse cardiovascular outcomes and higher incidence of extrahepatic malignancy [2, 3]. Among the analysed histological categories, the stage of liver fibrosis has repeatedly been demonstrated as the most important predictor not only of the liver-related but also overall mortality [3]. Interestingly, liver disease does not usually develop to the stage that would compromise overall survival, although live-related outcomes are worse in NAFLD accompanied by T2D as compared to nondiabetic counterparts [4].

For these reasons, active search for the presence and severity of NAFLD in patients with T2D seems intuitive but has not been endorsed by the most relevant international associations for diabetes yet. Possible reasons for this might be the lack of the effective treatment for NAFLD and reliable diagnostic tests [5]. As for the latter, liver biopsy is obviously not the method of choice given its invasiveness and high prevalence of NAFLD, whereas noninvasive diagnostic tests have not been completely evaluated in patients with T2D. Screening for the presence of liver fibrosis should be initiated at the primary care level among at-risk individuals by using simple biochemical tests (such as FIB-4), followed by the second batch tests (using direct markers of fibrosis or elastography) in case of indeterminate results [6]. However, assessment of liver fibrosis in NAFLD might be influenced by the amount of steatosis according to some reports, and the prognostic relevance of these noninvasive surrogates of liver disease in T2D patients has not been completely elucidated [7, 8].

Therefore, the aims of this study were to evaluate liver and nonliver-related outcomes in a real-life outpatient cohort of T2D, in relation to the prevalence and severity of NAFLD as assessed by noninvasive tests. Liver elastography and FIB-4 were tested for their diagnostic and prognostic performances in this cohort of patients.

## 2. Patients and Methods

### 2.1. Patients

This investigation was the combination of a cross-sectional study and longitudinal study. In cross-sectional part of the study, we analysed prevalence and severity of NAFLD by using FIB-4 and VCTE among patients with T2D. In the longitudinal part, recruited patients were followed until death or censored date in order to analyse their clinical outcomes in relation to these noninvasive indicators of liver fibrosis and steatosis.

Patients with T2D attending the outpatient diabetic clinic in the tertiary care hospital setting were prospectively assigned to noninvasive assessment of liver steatosis and fibrosis by vibration-controlled transient elastography (VCTE) by the FibroScan device. Three endocrinologists (DR, TM, and SM) referred the first 2 patients (out of around 25 patients having appointment at the respective day) showing up at the outpatient diabetic clinic working twice weekly in the morning from 1 August 2015 to 31 August 2018. Enrolment of the patients was not guided by any risk profiling from the medical history. During 37 months, 468 patients were referred to VCTE. At the diabetic clinic, all patients underwent standardized clinical and laboratory work-up as per the international guidelines [1]. Patients with a history of chronic liver disease of any aetiology other than NAFLD were excluded. In patients referred for the elastographic analysis who had elevated ALT, AST, or GGT, diagnostic work-up was performed in order to rule-out liver disease other than NAFLD (viral or autoimmune hepatitis, autoimmune cholangiopathy, alcoholic liver disease, Wilson's disease, haemochromatosis, and drug-induced liver injury). If any of these aetiologies was confirmed, the patient was excluded from the study.

The FIB-4 test was calculated based upon results of biochemistry determined from a blood sample drawn on the day of evaluation or within the last 3 months and according to the formula that consists of serum values of AST, ALT, platelets, and age of the patient (FIB-4 = (age (years) × AST (IU/L))/(platelets (10^9^/L) × ALT (IU/L)^1/2^)) [9]. FIB-4 cutoff values to rule-out (≤1.3) and rule-in (≥2.67) advanced fibrosis were used as suggested by the original study [9].

### 2.2. Assessment of Liver Fibrosis and Steatosis by Vibration-Controlled Transient Elastography

Liver stiffness measurement (LSM) as the surrogate for liver fibrosis and controlled attenuation parameter (CAP) for liver steatosis was assessed by VCTE with the FibroScan Touch 502 machine by 3 experienced operators (IG, SM, and TB, each having performed > 500 examinations) in fasting patients (at least for 3 hours, usually early in the morning after overnight fasting). The FibroScan probe (M or XL) was chosen according to the automatic probe selection tool embedded within the FibroScan machine. The probe was placed in the intercostal space over the right liver lobe usually in the anterior axillary line, in patient lying in the supine position with the right arm in the maximal abduction. Liver stiffness measurements were performed in the neutral breathing position, during a few seconds of apnoea. Ten LSM per patients were performed, and only those with IQR/median < 30% were considered reliable.

We used dichotomised LSM cutoff values to rule-out (<7.9 kPa) or to rule-in (≥9.6 kPa) advanced fibrosis as suggested by Wong VW et al. [10]. The presence of advanced fibrosis was chosen as the outcome of LSM because this stage of liver fibrosis has been demonstrated and widely accepted as the most relevant prognostic threshold associated with the accelerated development of morbidity and diminished survival in NAFLD [6, 11].

For the assessment of liver steatosis, controlled attenuation parameter (CAP) measurements were performed simultaneously with LSM by the FibroScan Touch 502 device. We used CAP cutoff values as reported by Karla's meta-analysis: 248 dB/m for *S* > 0, 268 dB/m for *S* > 1, and 280 dB/m for *S* > 2 [12]. Despite the reports that the accuracy of CAP declines when its IQR exceeded 40 dB/m, this has not been confirmed in the recent multicentric study, and therefore, we did not use this criterion as the indicator of reliability [13, 14].

### 2.3. Follow-Up

In a longitudinal extension of the study, patients were followed until death or censored date (31 December 2018) for the development of liver-related or any other morbidity or mortality by reviewing their medical history in the hospital database or by direct telephone contact with those who did not return for further controls.

Our primary outcome was mortality—liver or nonliver-related, whereas secondary outcome was morbidity, again liver-related and nonliver-related. We considered liver decompensation (jaundice, ascites, portohypertensive bleeding, or encephalopathy), development of hepatocellular carcinoma, or need for liver transplantation as liver-related morbidity. For nonliver-related morbidity, we considered cardiovascular events (acute coronary syndrome, stroke, coronary, or other vascular intervention), infection-related complications that required hospital admission, occurrence of any malignant tumour, and diabetes-related complications requiring hospitalisation (such as diabetic ketoacidosis or hyperosmolar syndrome).

### 2.4. Statistical Analysis

All statistical analysis procedures were performed using SPSS 24.0 (SPSS, Chicago, IL, USA). Standard parameters of descriptive statistics have been used for determination of baseline characteristics of all variables. All variables were evaluated for normal distribution by using the Kolmogorov–Smirnov test and Student's *t*-test with correction for unequal variances, where the appropriate was used in order to compare quantitative variables. The chi-square test was used to compare categorical variables. Pearson's or Spearman's nonparametric correlation used was appropriate. Kaplan–Meier curve with appropriate statistical measures was used to assess for survival. We used Cox regression to test the predictive potential of each observed variable for the survival. Variables found to be significant in univariate analysis were used to make multivariate analysis. A 95% level of significance for all tests was accepted for being important.

### 2.5. Ethical Issues

The study protocol conforms to the ethical guidelines of the 1975 Declaration of Helsinki (6th revision, 2008) as reflected in a priori approval by the institution's human research committee. Informed consent was obtained from each patient included in the study.

## 3. Results

We evaluated a total of 468 patients; in 14/468 (2.99%), VCTE measurements were unsuccessful, so a total of 454 patients with T2D (236; 52% males) with mean age (SD) of 62.5 (12) years were recruited. Baseline characteristics of included patients are provided in Table 1. The prevalence of liver steatosis and advanced fibrosis as assessed by CAP, LSM, and FIB-4 was 77.8%, 9.9%, and 3.1%, respectively. In multivariate analysis, factors independently associated with the risk of having advanced fibrosis (LSM ≥ 9.6 kPa) were AST (OR 1.057, 95% CI 1.035–1.080, *p* < 0.001) and cholesterol (OR 0.667, 95% CI 0.467–0.963, *p*=0.026). Liver steatosis as assessed by CAP did not have a significant impact on LSM (OR = 1.002, 95% CI = 0.997–1.007, *p*=0.45) readings; although significant but very weak correlation existed in Spearman's analysis (rho 0.189, *p* < 0.001). Independent risk factors for severe steatosis (CAP > 280 dB/m) were BMI (OR 1.093, 95% CI 1.045–1.143, *p* < 0.001), presence of arterial hypertension (OR 1.877, 95% CI 1.046–3.368, *p*=0.035), ALT (OR 1.029, 95% CI 1.011–1.048, *p*=0.002), and platelets (OR 0.996, 95% CI 0.992–1.000, *p*=0.043).

Comparison of the baseline characteristics between the patients with noninvasive indicators suggestive for the absence of advanced fibrosis (FIB-4 ≤ 1.3; LSM < 7.9 kPa) to those with higher values is presented in Table 2. Additionally, no significant difference existed (*p* > 0.05) in hypertension prevalence and statin use between subgroups presented in Table 2. However, higher frequency of males (96/223; 43.0%) vs. females (67/209: 32.1%) was detected in the subgroup of patients with FIB-4 over 1.3 (*p*=0.024). Also, higher frequency of XL probe (71/321; 22.1%) vs. M probe (15/133; 11.3%) use for VCTE examination was detected in the subgroup of patients with LSM≥7.9 kPa (*p*=0.023).

### 3.1. FIB-4 Score as a Triage Tool with VCTE Serving as the Reference Method

Since the prevalence of advanced fibrosis clearly differed with respect to the noninvasive method used (9.9% by LSM vs. 3.1% by FIB-4), we further explored their interrelationship. We decided to use VCTE as the reference method because it was demonstrated to have much less indeterminate or misclassified cases for advanced fibrosis as compared to FIB-4 (27% vs. 58%). [15] Among 433 patients with available data, FIB-4 values ranged 0.13–7.94 with median of 1.16 (IQR: 0.84–1.53). In 269 (62.1%) patients, FIB-4 was ≤1.3, whereas it was ≥2.67 in only 14 (3.1%) patients (Table 1). In patients with FIB-4 ≤ 1.3, there was 224/269 (83.6%) with VCTE < 7.9 kPal; whereas in patients with FIB-4 > 1.3, there was 37/164 (22.6%) patients with VCTE ≥ 7.9 kPa. More interestingly, among 269 patients with FIB-4 ≤ 1.3, 24 (8.9%) had LSM ≥ 9.6 kPa indicative of advanced fibrosis, and 13 (4.8%) had LSM ≥ 11.5 kPa indicative of cirrhosis; whereas in patients with FIB-4 value > 1.3, there were 21/164 (14.7%) with LSM ≥ 9.6 kPa. As expected, the overall agreement between FIB-4 and VCTE was not statistically significant when assessed with kappa statistics (*κ* = 0.065; *p*=0.133).

Diagnostic performance of the FIB-4 test at the threshold value of 1.3 for advanced (F3) fibrosis as defined by LSM 9.6 kPa in our sample with the prevalence of advanced fibrosis of 10.3% was 46.7% sensitivity, 63.4% specificity, 12.8% PPV, 91.9% NPV, 1.28 LR+, and 0.84 LR−. The AUROC for FIB-4 and for predicting LSM ≥ 9.6 kPa was 0.639, 95% CI = 0.545–0.733, *p*=0.004. In order to explore if lowering the FIB-4 cutoff value would have improved its diagnostic performance, i.e., decrease the proportion of false-negative patients with advanced fibrosis as determined by VCTE, we chose 1.1 cutoff having 94% NPV in AUROC analysis. However, even with this FIB-4 cutoff, still 11/189 (5.8%) patients had LSM ≥ 9.6 kPa. At this threshold, FIB-4 had sensitivity 73.8%, specificity 45.5%, PPV 12.7%, NPV 94.2%, LR+ 1.35, and LR− 0.58 for advanced fibrosis.

### 3.2. Survival of Patients in 2 Years Follow-Up

**Figure 1:**
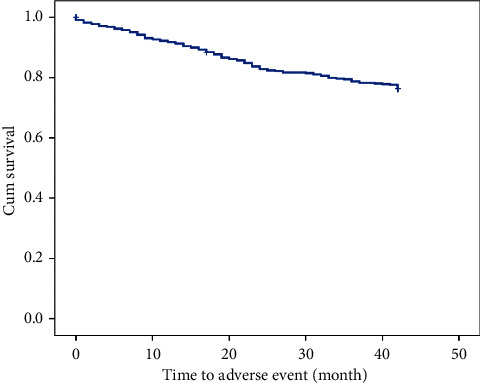
Kaplan–Meier curve of overall survival—time to occurrence of any adverse event during the follow-up period.

During the median follow-up time of 25 months (IQ range: 9–39), a total of 106 (23.3%) patients experienced an adverse event: cardiovascular in 50 (11%) patients, infection-related in 31 (6.8%), diabetes-related in 22 (4.8%), and oncological in 16 (3.5%), whereas there were no liver-related complications. Seventeen (3.7%) patients died during the follow-up (all deaths nonrelated to liver disease). A Kaplan–Meier curve of overall survival until any adverse event is shown in Figure 1. Mean time to any adverse event was 36.5 months (95% CI: 35.4–37.5).

We selected a subgroup of patients with the follow-up period of 24 months and more (*n* = 374). A total of 33 patients experienced any adverse event (8.8%): cardiovascular in 17 (4.5%), infection-related in 11 (2.9%), diabetes-related in 7 (1.9%), oncological in 3 (0.8%), and again without liver-related complications. There were 4 deaths (1.1%) in this subgroup of patients, again all nonrelated to liver disease. Mean time to any adverse event was 41 months (95% CI: 40.9–41.5).

### 3.3. Predictors of Morbidity and Mortality in 2 Years Follow-Up

We performed a univariate Cox regression analysis for the adverse outcome (occurrence of any morbidity or mortality) with all the variables of interest as possible predictors (Table 3). Age, FIB-4, AST, and platelets (PLT) count were significant predictors of adverse outcomes, with borderline significance for CAP and ALT.

The possible influence of different CAP categories on the composite outcome was additionally analysed. Interestingly, the best outcomes in terms of morbidity were observed in the group with most severe steatosis (*X*^2^ = 9.03, d*f* = 3, *p*=0.029) (Table 4), whereas no difference in terms of mortality existed (*p*=0.128). Due to small number of patients in groups with S1 and S2 steatosis, which might have influenced these results, we divided the entire sample into 2 groups according to CAP values ≤280 dB/m and >280 dB/. In Cox regression analysis, we found no effect of CAP at this threshold on survival (HR = 0.85, 95% CI = 0.31–2.29, *p*=0.75) or occurrence of any morbidity (HR = 0.73, 95% CI = 0.48–1.09, *p*=0.12).

Variables found to be significant predictors in univariate analysis were additionally included and analysed with stepwise multivariate Cox regression, and only age (HR = 1.046, 95% CI = 1.026–1.066, *p*=0.003) and platelets count (HR = 1.003; 95% CI = 1.001–1.06; *p*=0.016) were found to be significant predictors of any morbidity and mortality, while FIB-4 (*p*=0.10) and AST (*p*=0.64) were not. This was also true for Cox analysis regarding the mortality—again, age (HR = 1.12, 95% CI = 1.06–1.19, *p*=0.002) and higher platelet count (HR = 1.007, 95% CI = 1.000–1.013, *p*=0.037) were significant predictors of mortality, while FIB-4 (*p*=0.28) and AST (*p*=0.47) were not.

**Figure 2:**
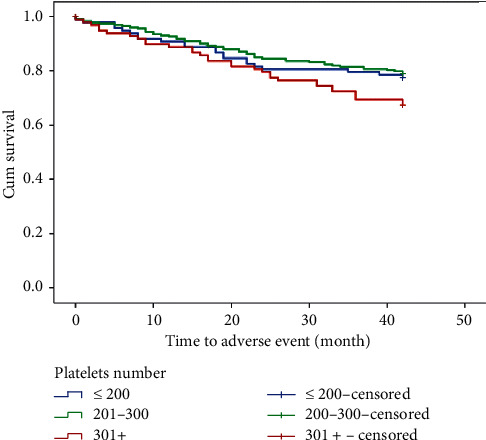
Kaplan–Meier curve of overall survival—time to occurrence of any adverse event during the follow-up period stratified according to platelets number.

Then, we divided the sample to three subgroups: into those with platelet count <200 (98; 22.6%), 201–300 (236; 54.4%), and >300 (100; 23.0%) × 10^9^/L, whereas for 20 patients (4.4%), platelet count was not available. The sample was then stratified via Kaplan–Meier analysis according to above categories, and although the difference between categories according to survival was not significant (*p*=0.08), the borderline significance suggests the tendency for higher morbidity and mortality in patients with higher platelet count (Figure 2).

## 4. Discussion

This study conducted over the large cohort of patients with T2D reveals high prevalence of overweight/obesity and liver steatosis (both around 80%). Results of FIB-4 and VCTE were not concordant in predicting the proportion of patients with/without advanced fibrosis. Over the follow-up period of median 2 years, no liver-related morbidity or deaths were reported, and therefore, the real prevalence of advanced fibrosis in this cohort was likely low and overestimated by LSM ≥ 9.6 kPa. Among 23% of patients who experienced adverse outcome, half was caused by cardiovascular events. FIB-4, LSM, and CAP as the noninvasive surrogates of fibrosis and steatosis, respectively, were not predictive for adverse outcomes in the analysed cohort and the period of time.

Diabetes is a very prevalent condition, affecting around 9% of the world adult population [1] and goes hand-by-hand with the epidemics of overweight/obesity. In Europe, around 50% of population is overweight, and almost half of that number is obese [15]. Obesity and the resultant insulin resistance are the important metabolic conditions associated with the development of NAFLD, although several authors argue pointing to the more important pathophysiological role of the fatty liver that facilitates development of insulin resistance and T2D [16]. Whichever is right, people with T2D, especially with obesity and NAFLD share common dysfunction of metabolic pathways and are accompanied by other comorbidities such as dyslipidemia and arterial hypertension, commonly known as metabolic syndrome [17].

This syndrome is associated with increased cardiovascular morbidity and mortality. Patients with fatty liver have relatively good prognosis, and the major determinant of their long-term outcome is the presence of liver fibrosis [2]. Around 1/3 of patients with NAFLD develop fibrosis and are in risk for liver-related morbidity and mortality [18]. Also, these patients are more endangered in terms of cardiovascular and oncological events and mortality [3]. It has been repeatedly shown that the presence of T2D in patients with NAFLD represents risk for progressive course of liver disease, and for vice versa, some conflicting results were published [4, 19].

These are the reasons why we should be interested at evaluating patients with T2D for the presence and severity of NAFLD. For this purpose, noninvasive tests have gained much popularity for being easy to perform, available, painless, and with acceptable accuracy in diagnosing and quantifying liver steatosis and fibrosis. Whereas, the impact of steatosis has not been proven, and fibrosis plays the prominent role on the development of liver-related complications as well as overall morbidity and mortality as already pointed out. According to recent data, steatosis might be present even in the patients with the compensated advanced chronic liver disease (cACLD), and the higher grade of steatosis might be associated with the worse prognosis in terms of decompensation and death [20–22]. For less advanced stages of chronic liver disease, probably the rationale for quantifying liver steatosis is to objectively follow reduction in steatosis while the patient is taking lifestyle measures to correct his/her metabolic abnormalities.

Our cohort of T2D is similar to the other cohorts reported in the literature. Around 80% of them are overweight/obese and 80% has NAFLD, and almost 10% of them have advanced fibrosis according to LSM assessment by VCTE [23]. However, real proportion of advanced fibrosis would have probably been lower if it was assessed histologically, since it has been previously demonstrated that VCTE had only 59% PPV, meaning that at most 6% of our cohort would in fact have advanced fibrosis [23]. This conclusion is furtherly supported by the absence of liver-related events in our cohort during the follow-up. The potential influence of steatosis on LSM readings is rather controversial issue as some reports do and the others do not suggest association between them [7, 8, 14]. Although a weak correlation between CAP and LSM existed, in multivariate analysis, CAP was not independently associated with the risk of having advanced fibrosis in our cohort.

Based on our data, FIB-4 < 1.3 has 92% NPV for ruling out advanced fibrosis in patients with T2D, with marginal improvement of NPV to 94% at lower FIB-4 threshold of 1.1. Our results are in keeping with current evidences claiming high NPV of the similar order of magnitude for FIB-4, but its PPV is suboptimal, and in addition to this, significant number of false-negative cases (8% according to our results) still appears below this threshold [24].

In terms of predictive capability of baseline noninvasive parameters, only age and higher platelets count were predictive for adverse outcomes in our cohort, whereas other demographic, biochemical (including FIB-4), or elastographic (LSM and CAP) values were not. Our results are in agreement with recently published data from Edinburgh cohort of T2D patients demonstrating suboptimal predictive ability of several noninvasive biochemical indices including FIB-4 which had 11–18% false-negative predictive rate for cirrhosis or HCC at 1.3 cutoff, whereas PPV of 40–46% at 2.67 cutoff value was equally poor [25]. Similarly, LSM did not influence the outcomes, although 10% of patients had liver stiffness reading over the threshold for advanced fibrosis (≥9.6 kPa). However, VCTE in general has much better performances to rule-out than rule-in advanced fibrosis or cirrhosis. Published PPVs for advanced fibrosis at LSM threshold of 9.6 or 9.7 kPa ranges 59–72.4%, whereas PPV for cirrhosis defined at cutoff 11.5 kPa was below 50% in Wong's study and for cutoff 13.6 kPa only 29% in Eddowes' study [10, 14, 23]. In the latter study, optimised cutoff for cirrhosis with 90% specificity was 20.9 kPa, and even at this high threshold, its PPV was only 37%. Therefore, LSM ≥ 9.6 kPa likely overestimated real prevalence of advanced fibrosis in our cohort. Furthermore, only 5 patients had LSM values over 20.9 kPa, and given the low PPV, it might be that in fact no patient had cirrhosis. In addition to probably very small proportion of patients with advanced fibrosis, our results are also not surprising because the follow-up period was relatively short. Bearing in mind that development of liver fibrosis and end-stage liver disease is relatively a slow process, it is not unexpected that no liver-related adverse outcomes were noticed. This may lead to general conclusion that noncirrhotic patients with T2D might be relatively safely followed by VCTE every 2 years. This is in line with the results of the Swedish study on the natural history of NAFLD (from general population, not only diabetics) which demonstrated that it needs at least 2.3 years for the first 10% of patients with advanced liver fibrosis to develop cirrhosis, liver decompensation, or HCC [11]. However, the presence of cirrhosis, when reliably diagnosed, should lead to intensified surveillance for the occurrence of HCC every 6 months by ultrasound according to current recommendations [26]. As for the predictive role of platelets count for the CV morbidity/mortality, this association has already been demonstrated and probably results from higher thrombogenic risk in patients with higher platelet count [27].

This study has limitations. First of all, patients were prospectively included over the long period of time, whereas the follow-up period was relatively short, so we were not able to analyse neither long-term outcomes of patients with T2D and NAFLD nor the potential impact of LSM, CAP, or FIB-4 in this regard. Furthermore, this study lacks liver biopsy to make firm conclusions about the severity of liver disease and the interrelationship between some histological categories and their influence on CAP and LSM. Nevertheless, outcomes were clearly defined and analysed as the occurrence of liver-related or any other morbidity and mortality. There is also an issue of LSM threshold values for various fibrosis grades and current controversy whether the use of the XL probe or CAP value has an impact on LSM measurement. Given the recent evidence, neither the probe type (M/XL) nor the CAP value has been confirmed to influence LSM as assessed by VCTE [14]. Which is the best cutoff value for a certain stage of liver fibrosis may be a matter of discussion because there is no 100% agreement between the studies and authors. We used cutoff values proposed by Wong et al. because most studies published so far referred to these cutoff values [9]. We do not believe that using the different cutoffs would likely change the main messages derived from this research.

In conclusion, T2D patients in this cohort had high prevalence of overweight/obesity and liver steatosis (both around 80%). In this group of patients, FIB-4 as a triage tool has good NPV for ruling-out advanced fibrosis, with marginal improvement at the lower threshold of 1.1. Real prevalence of advanced fibrosis in our cohort was likely low and overestimated by LSM ≥ 9.6 kPa by VCTE. This conclusion is supported by the absence of liver-related events during the follow-up period. Therefore, in the cohort of patients with T2D with probably low prevalence of advanced fibrosis, noninvasive tests for fibrosis were not predictive for adverse outcomes over the analysed period of time, and the same holds truth for the prognostic impact of liver steatosis quantified noninvasively by CAP. Among 23% of patients who experienced adverse outcome, half was caused by cardiovascular events. Patients with T2D could probably be safely monitored for liver-related complications in 2 years intervals, provided that cirrhosis has been reliably ruled-out.

## Figures and Tables

**Table 1:** Baseline characteristics of patients.

Variable	*N* = 454, median (IQR)/*n* (%)
Age (years)	64 (56–71)
Male	236 (52%)
BMI (kg/m^2^)	30.09 (26.45–34.34)
BMI < 25 (kg/m^2^)	79 (17.4%)
BMI 25–30 (kg/m^2^)	146 (32.2%)
BMI > 30 (kg/m^2^)	229 (50.4%)
AST (IU/L)	22 (18–28)
ALT (IU/L)	24 (18–36)
GGT (IU/L)	29 (20–49)
ALP (IU/L)	71 (60–90)
PLT (×10^9^/L)	245 (206–295)
TGL (mmol/L)	1.7 (1.2–2.5)
CHOL (mmol/L)	4.7 (4.0–5.6)
HDL (mmol/L)	1.2 (1.0–1.4)
LDL (mmol/L)	2.7 (2.1–3.5)
HbA1C (mmol/mol)	59 (50–76)
Hypertension	328 (72.2%)
Statin use (*N* = 448)	223 (49.1%)
Skin capsular distance (cm)	2.16 (1.80–2.51)
Use of XL probe	321 (70.7%)
VCTE (kPa)	5.6 (4.4–7.1)
VCTE ≤ 7.9 kPa	368 (81.1%)
VCTE > 7.9 kPa	86 (18.9%)
VCTE ≥ 9.6 kPa	45 (9.9%)
VCTE ≥ 11.5 kPa	33 (7.3%)
CAP (dB/m) (*N* = 453)	310 (256–347)
No steatosis (≤248 dB/m)	101 (22.2%)
Steatosis gr. I (249–268 dB/m)	29 (6.4%)
Steatosis gr. II (269–280 dB/m)	22 (4.8%)
Steatosis gr. III (>280 dB/m)	302 (66.5%)
FIB-4 (*N* = 433)	1.16 (0.84–1.53)
FIB-4 ≤ 1.3	269 (62.1%)
FIB-4 ≥ 2.67	14 (3.1%)

VCTE, vibration-controlled transient elastography; CAP, controlled attenuation parameter; BMI, body mass index; CHOL, total cholesterol; TGL, triglycerides; PLT, platelets.

**Table 2:** Comparison of clinical and biochemical characteristics of included patients according to FIB-4 and VCTE values.

	FIB-4	*N*	Mean	SD	*p* value	VCTE	*N*	Mean	SD	*p* value
Age	≤1.3	269	59.61	12.10	<0.001	<7.9 kPa	368	62.38	12.24	0.57
	>1.3	164	66.93	10.33		≥7.9 kPa	86	63.20	10.70	
VCTE (kPa)	≤1.3	269	6.26	3.46	0.04	<7.9 kPa	368	5.17	1.28	<0.001
	>1.3	164	7.04	4.29		≥7.9 kPa	86	12.35	5.16	
CAP (dB/m)	≤1.3	269	301.28	63.36	0.35	<7.9 kPa	368	295.11	66.13	0.024
	>1.3	164	295.07	71.61		≥7.9 kPa	86	312.85	64.54	
BMI (kg/m^2^)	≤1.3	268	30.87	7.27	0.09	<7.9 kPa	368	30.24	6.89	0.33
	>1.3	164	29.71	6.64		≥7.9 kPa	85	31.07	7.72	
HbA1C	≤1.3	269	66.30	22.48	0.004	<7.9 kPa	347	63.39	21.48	0.36
	>1.3	157	60.07	19.78		≥7.9 kPa	82	65.82	22.28	
AST	≤1.3	269	21.60	7.92	<0.001	<7.9 kPa	359	23.80	10.98	<0.001
	>1.3	164	36.09	33.55		≥7.9 kPa	86	40.76	44.36	
ALT	≤1.3	269	28.79	18.25	<0.001	<7.9 kPa	359	28.96	21.12	<0.001
	>1.3	164	39.16	41.81		≥7.9 kPa	86	47.69	50.00	
GGT	≤1.3	266	38.86	34.64	<0.001	<7.9 kPa	355	40.26	43.14	<0.001
	>1.3	164	68.39	117.28		≥7.9 kPa	86	95.26	149.04	
ALP	≤1.3	229	76.03	28.74	0.68	<7.9 kPa	307	74.26	27.23	<0.001
	>1.3	147	77.35	29.78		≥7.9 kPa	75	87.07	37.07	
PLT	≤1.3	269	279.70	64.64	<0.001	<7.9 kPa	352	253.37	70.15	0.66
	>1.3	164	208.79	55.99		≥7.9 kPa	82	249.62	72.12	
TGL	≤1.3	254	2.18	1.62	0.32	<7.9 kPa	346	2.40	5.58	0.74
	>1.3	159	2.70	8.07		≥7.9 kPa	79	2.19	1.86	
CHOL	≤1.3	254	4.88	1.35	0.202	<7.9 kPa	346	4.79	1.33	0.57
	>1.3	159	4.71	1.31		≥7.9 kPa	78	4.88	1.38	
HDL	≤1.3	236	1.26	0.71	0.85	<7.9 kPa	317	1.29	0.65	0.006
	>1.3	144	1.28	0.39		≥7.9 kPa	74	1.14	0.36	
LDL	≤1.3	222	2.85	1.13	0.14	<7.9 kPa	294	2.75	1.10	0.32
	>1.3	133	2.67	1.06		≥7.9 kPa	71	2.89	1.10	

VCTE, vibration-controlled transient elastography; CAP, controlled attenuation parameter; BMI, body mass index; CHOL, total cholesterol; TGL, triglycerides.

**Table 3:** Univariate Cox regression analysis of predictors of occurrence of adverse events during the follow-up period.

	HR	95.0% CI for HR		*p* value
		Lower	Upper	
Gender	1.43	0.85	2.41	0.17
Age	1.04	1.01	1.08	**0.02**
BMI (kg/m^2^)	1.00	0.97	1.04	0.81
VCTE (kPa)	0.99	0.91	1.07	0.78
CAP (dB/m)	1.00	0.99	1.00	0.05
FIB-4	2.63	1.08	6.39	**0.03**
Hypertension	1.44	0.76	2.74	0.27
Statin	1.35	0.80	2.28	0.26
Smoking	1.84	0.96	3.54	0.07
HbA1C	1.00	0.99	1.02	0.51
AST	0.96	0.92	1.00	**0.04**
ALT	1.02	1.00	1.04	0.05
GGT	1.00	0.99	1.00	0.46
ALP	1.00	0.99	1.01	0.72
PLT	1.01	1.00	1.01	**0.03**
TGL	0.91	0.76	1.10	0.35
CHOL	1.38	0.81	2.35	0.24
HDL	0.70	0.31	1.54	0.37
LDL	0.85	0.48	1.51	0.58

Statistically significant values (*p* < 0.05) are depicted in bold format. VCTE, vibration-controlled transient elastography; CAP, controlled attenuation parameter; BMI, body mass index; CHOL, total cholesterol; TGL, triglycerides.

**Table 4:** Influence of the CAP value on composite outcomes (any morbidity or mortality).

			Any morbidity or mortality		Total
			No	Yes	
CAP value (dB/M)	≤248	*N*	67	33	100
		%	67.0%	33.0%	100.0%
	249–268	*N*	21	8	29
		%	72.4%	27.6%	100.0%
	269–280	*N*	15	7	22
		%	68.2%	31.8%	100.0%
	>280	*N*	242	58	300
		%	80.7%	19.3%	100.0%
Total		*N*	345	106	451
		%	76.5%	23.5%	100.0%

*X* ^2^ = 9.03; d*f* = 3; *p*=0.029. CAP, controlled attenuation parameter.

## Data Availability

The data used to support the findings of this study are available from the corresponding author upon request.

## Additional Points

- Real prevalence of advanced fibrosis in our patients with T2D seems low
- LSM ≥ 9.6 kPa by VCTE likely overestimates advanced fibrosis in patients with T2D
- No liver-related morbidity/mortality occurred over 2 years
- FIB-4, LSM, and CAP were not predictive for adverse outcomes
- Liver fibrosis may be safely reassessed in 2 years in noncirrhotic T2D patients
